# Vibration-induced postural reactions: a scoping review on parameters and populations studied

**DOI:** 10.3389/fnhum.2023.1307639

**Published:** 2024-01-03

**Authors:** Michaël Bertrand-Charette, Marie-Pier Perron, Rubens A. da Silva, Louis-David Beaulieu

**Affiliations:** ^1^BioNR Research Lab, Université du Québec à Chicoutimi, Saguenay, QC, Canada; ^2^Département des Sciences de la Santé, Centre intersectoriel en santé durable, Université du Québec à Chicoutimi (UQAC), Saguenay, QC, Canada; ^3^Centre Intégré de Santé et Services Sociaux du Saguenay—Lac-Saint-Jean (CIUSSS SLSJ), Specialized Geriatrics Services–La Baie Hospital, Saguenay, QC, Canada

**Keywords:** vibration, proprioception, postural reactions, vibration parameters, methodological rationale

## Abstract

**Objective:**

Mechanical vibration is an effective way for externally activating Ia primary endings of the muscle spindles and skin mechanoreceptors. Despite its popularity in proprioception and postural control studies, there is still no review covering the wide variety of vibration parameters or locations used in studies. The main purpose of this scoping review was thus to give an overview of general vibration parameters and to identify, if available, the rationale for justifying methodological choices concerning vibration parameters.

**Methods:**

Three databases (Pubmed, CINHAL, and SPORTDiscus) were searched from inception to July 2022. Included articles were to focus on the study of muscle spindles and skin mechanoreceptors vibration in humans and assess postural control. Following inclusion, data regarding demographic information, populations, vibration parameters and rationale were extracted and summarized.

**Results:**

One hundred forty-seven articles were included, mostly targeting lower extremities (*n* = 137) and adults (*n* = 126). The parameters used varied widely but were most often around 80 Hz, at an amplitude of 1 mm for 10–20 s. Regarding rationales, nearly 50% of the studies did not include any, whereas those including one mainly cited the same two studies, without elaborating specifically on the parameter's choice.

**Conclusion:**

This scoping review provided a comprehensive description of the population recruited and parameters used for vibration protocols in current studies with humans. Despite many studies, there remain important gaps of knowledge that needs to be filled, especially for vibration amplitude and duration parameters in various populations.

## 1 Introduction

Mechanical vibration of muscles, tendons and skin has been widely used as an effective way for externally activating Ia primary endings surrounding the non-contractile central portions of the muscle spindles and skin mechanoreceptors. This approach is further used to assess proprioception (i.e., sense of joint movement and position), motor and postural control (Roll and Vedel, 1982; Kavounoudias et al., 1999, 2001; Kadri et al., 2020). In fact, the proprioceptive role of muscle spindles and cutaneous mechanoreceptors has been studied with vibration for more than 50 years (Goodwin et al., 1972; Burke et al., 1976; Roll and Vedel, 1982; Kavounoudias et al., 2001). In the absence of vision, mechanical vibration of a superficial tendon generates an illusion of movement coherent with the stretch of the vibrated muscle (Roll and Vedel, 1982; Roll et al., 1989). When applied during upright posture, it can also elicit postural reactions (VIB-induced postural reactions or VIB-PR) consistent with the postural function of the targeted receptors. For example, bilateral vibration of Achilles tendons or forefoot soles sends a false sensory information of forward leaning, as if calf muscles were stretched or more body pressure was put toward the front of the feet, respectively (Kavounoudias et al., 1999, 2001). In the presence of normal postural and sensorimotor control networks, a quick backward postural reaction is observed in response to the sensory disturbance (Kavounoudias et al., 1999, 2001).

Vibration-induced effects were so far studied using various biomechanical and neurophysiological tools. Microneurographic recordings of nerve fibers provided evidence on the types of somatosensory fibers that are preferentially activated when using tendon or cutaneous vibration (Burke et al., 1976; Roll and Vedel, 1982; Ribot-Ciscar et al., 1989; Inglis et al., 2002). Others rather investigated the biomechanical characteristics of VIB-PR based on measurements obtained by a force platform, such as the center of pressure (CoP) (Busquets et al., 2018; Baudry and Duchateau, 2020; Kadri et al., 2020; Oku et al., 2020), center of mass/gravity (CoM/CoG) (El-Kahky, 2000; Yagi et al., 2000; Maurer et al., 2001; Keshner et al., 2014; Mullie and Duclos, 2014; Cyr et al., 2019), or using 3D kinematic analysis systems (Smiley-Oyen et al., 2002; Ribot-Ciscar et al., 2004; Thompson et al., 2011; Mullie and Duclos, 2014). Evidence supports that Ia fibers from muscle spindle endings and slowly adapting cutaneous receptors are more sensitive to vibration and likely responsible for VIB-PR (Roll and Vedel, 1982; Vedel and Roll, 1982; Ribot-Ciscar et al., 1989; Kavounoudias et al., 1999, 2001; Proske and Gandevia, 2009). Then, research over the last decades mostly focused on comparing VIB-PR between different populations to further explore their diagnostic/therapeutic potential (El-Kahky, 2000; Bonan et al., 2015; Caccese et al., 2021).

However, there is still no published guideline covering the key methodological aspects to consider when using the VIB-PR paradigm. Methods and vibration parameters greatly vary across studies, which makes it difficult to compare results within the literature and draw adequate conclusions. There are several parameters known to directly influence vibration-induced effects and postural reactions, such as the location of the vibrator and vibration's frequency, amplitude or duration (Taylor et al., 2017; Beaulieu et al., 2020). In particular, it has been shown that muscle spindle afferents are more strongly activated at vibration frequencies around 70–80 Hz (Roll and Vedel, 1982; Kavounoudias et al., 2001; Taylor et al., 2017), although many studies have used frequencies as low as 0.28 Hz (Caccese et al., 2020, 2021) and up to 250 Hz (El-Kahky, 2000). Too much heterogeneity and lacking consensus significantly affect the overall impact of VIB-PR research.

Some previous reviews on vibration applications (Taylor et al., 2017; Aboutorabi et al., 2018) focused on particular population such as older people (Aboutorabi et al., 2018), on a specific body segment (Jamal et al., 2020) or on non-postural applications of vibration such as the renowned vibration-induced kinesthetic illusion paradigm (Taylor et al., 2017). Of note, “whole-body vibration” should not be confounded with focal vibration used for VIB-PR and kinesthetic illusions, as it uses a different technology and does not target the same mechanisms and physiological functions [cf. reviews on the topic of whole-body vibration (Lings and Leboeuf-Yde, 2000; Rogan et al., 2017)]. VIB-PR literature is thus filled with good pieces of information, but no review covered the wide variety of vibration parameters or locations used so far.

The main purpose of this scoping review was thus to give an overview of general vibration parameters (location, frequency, amplitude, duration) used so far to describe the current state of evidence and identify the most important knowledge gaps and future opportunities. A second purpose was to identify, if available, the rationale used by the authors for justifying their methodological choices concerning vibration parameters. It should be mentioned that the effect of VIB-PR will not be covered in the present scoping review and should therefore be addressed in future studies.

## 2 Materials and methods

### 2.1 Study design and methodological framework

Scoping reviews have been described as studies aiming “to map rapidly the key concepts underpinning a research area and the main sources and types of evidence available, and can be undertaken as stand-alone projects in their own right, especially where an area is complex or has not been reviewed comprehensively before” (Arksey and O'Malley, 2005; Joanna Briggs Institute, 2015). Therefore, the scoping review was the appropriate choice to review the extent and range of the different studies using vibration-induced postural reactions in human research. This particular method of analysis will be useful to determine the value of undertaking a full systematic review and to identify research gaps in the existing literature regarding VIB-PR in humans (Arksey and O'Malley, 2005). The review protocol was thus based on the methodological framework suggested by Arksey and O'Malley (2005).

### 2.2 Identification and selection of studies

Three databases (Pubmed, CINHAL, and SPORTDiscus) were searched from inception to July 2022. A combination of keywords (with or without truncation), controlled vocabulary thesaurus and Boolean operators were used for each database. The search terms for Pubmed were: Vibrat^*^ AND (postur^*^ OR balance OR equilibr^*^ OR “Postural Balance” [Mesh]) AND (tend^*^ OR “Tendons” [Mesh] OR cutan^*^ OR “Mechanoreceptors” [Mesh]) and was then adapted for each database. This led to the following keywords for CINHAL: Vibrat^*^ AND (MH “Balance, Postural” OR postur^*^ OR balance OR equilibr^*^) AND (tend^*^ OR cutan^*^ OR mechanoreceptor). Finally, the search terms for SPORTDiscus were: Vibrat^*^ AND (postur^*^ OR balance OR equilibr^*^) AND (tend^*^ OR cutan^*^ OR mechanoreceptor). Finally, a manual search was performed in reference lists of the included studies to include articles that were not found by the standard literature search in the three databases. To be included, the articles had to (1) study muscular, tendinous, or cutaneous vibration, (2) in humans, and (3) to assess postural control. Articles were excluded if the protocols were focused on (1) whole body vibration or (2) vibration platforms. The language of the articles was limited to French and English. The literature search was performed by two evaluators (MBC and MPP) who individually screened the titles and abstracts before selecting the articles included.

### 2.3 Data extraction and summary of data

Data extraction was performed by MBC and MPP using a standardized extraction grid custom-made for this scoping review. Data related to demographic information, as well as the different populations included in each article, were extracted. Regarding data specific to vibration parameters (primary objective), information on location, duration, frequency, and amplitude have been extracted. Regarding data summary, qualitative data were pooled and presented according to their frequency, while quantitative data were extracted, combined, and described using descriptive statistics (means and standard deviations). Regarding the secondary objective, data for the rationale behind the vibration parameters (including citations to other studies) were extracted from the methodology section, when available. If the rationale was not presented in the methodology section, the introduction was screened for reference regarding the rationale.

## 3 Results

**Figure 1:**
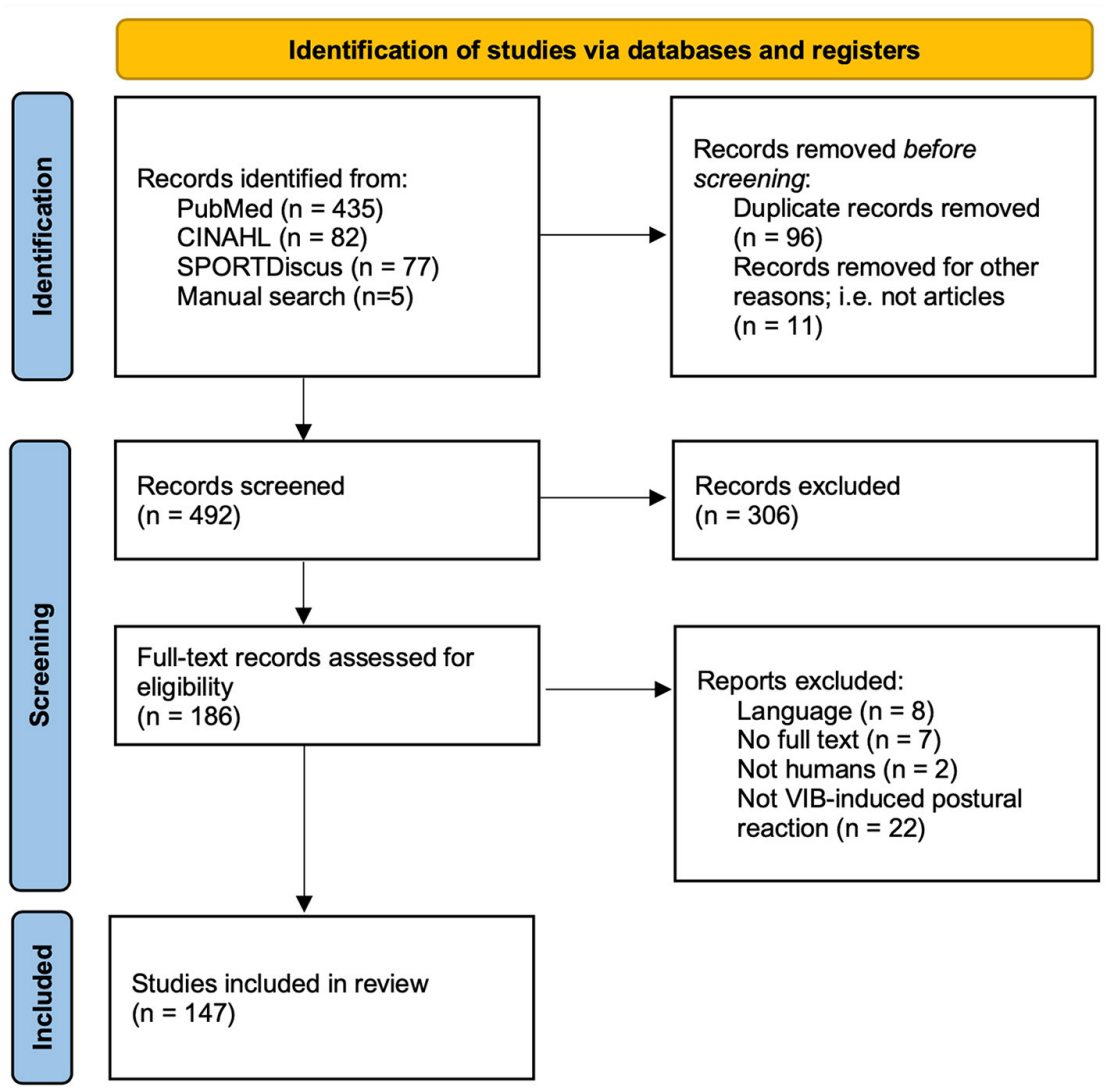
Adaptation of the PRISMA 2020 flow diagram for new systematic reviews. Adapted from Page et al. (2021).

Out of the 599 articles obtained after the literature search in the three databases and by manual search, 186 were assessed for eligibility. Following full-text reviews, 147 studies were included in the present review (Figure 1). Descriptive information of the population and vibration parameters for all 147 included studies can be found in Supplementary Data Sheet S1, while data regarding rationale can be found in Supplementary Data Sheet S2.

### 3.1 Population studied

**Figure 2:**
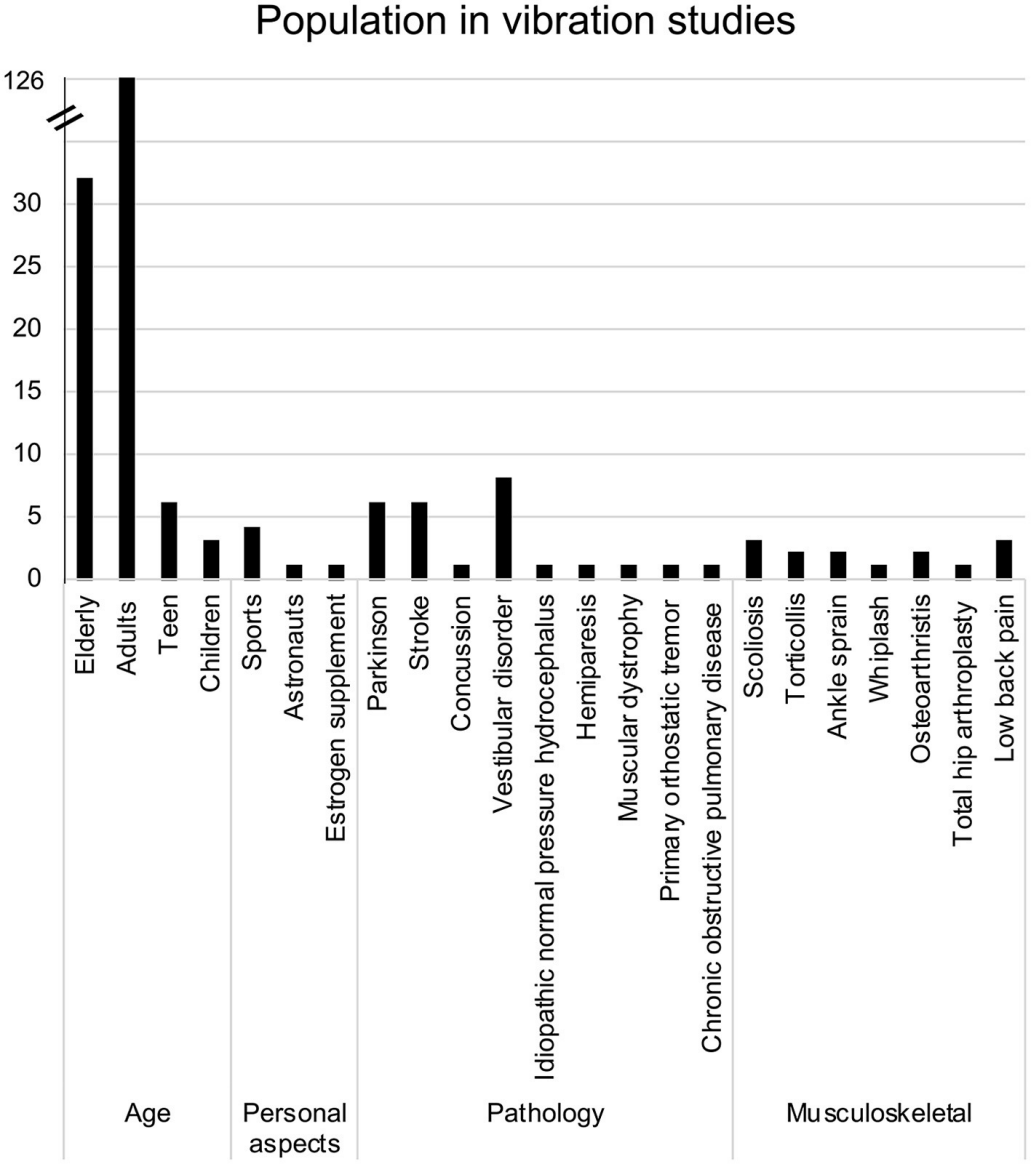
Bar graph of the frequency of populations recruited in vibration studies.

As presented in Figure 2, the majority of studies recruited adults (*n* = 126 studies) in at least one of the tested groups. In most cases, only adults were recruited as participants. However, in 20 studies, adults were compared to the older adults, which was the second most common group of participants across the included studies (*n* = 32). Individuals were classified as “older adults” if the mean age of the group was over 65 (Orimo et al., 2006; Sabharwal et al., 2015). The mean age of participants across all studies was 39.8 ± 20.6 years old. When separating participants according to their respective age groups (as identified in the studies), the mean age for adults was 32.3 ± 12.7 years, 70.8 ± 5.2 years for the older adults, while children and teens were respectively 8.9 ± 1.8 and 15.2 ± 1.5 years old. Overall, a total of 1,666 males and 1,608 females were recruited, with some recruiting only males (Hayashi et al., 1981, 1988; Roll et al., 1998; Vuillerme et al., 2001; Blouin et al., 2003; Stolbkov and Orlov, 2009; Kanakis et al., 2014; Billot et al., 2015; Ema et al., 2020) and others only females (Naessen et al., 1997; Spiliopoulou et al., 2012; Maitre et al., 2013a,b, 2014; Mahmoudian et al., 2016; Fortin et al., 2019; Oku et al., 2020).

Apart from age, three other subgroups were identified (Figure 2). The first subgroup, called “Personal aspects,” comprises studies that have tested participants presenting characteristics relative to sports (Vuillerme and Cuisinier, 2008; Busquets et al., 2018; Caccese et al., 2020, 2021), work [astronaut (Roll et al., 1998)] and medication intake [estrogen supplements (Naessen et al., 1997)]. The second and third subgroups include participants with various neurological, cardiovascular or musculoskeletal conditions. However, most specific characteristics within the three subgroups were studied by fewer than five papers, except for Parkinson's disease (Smiley-Oyen et al., 2002; Valkovič et al., 2006; Vaugoyeau et al., 2011; Bekkers et al., 2014; Hwang et al., 2016; Pereira et al., 2016), stroke survivors (Lin et al., 2012; Bonan et al., 2013, 2015; Duclos et al., 2015; Jamali et al., 2019; Sajedifar et al., 2020) and vestibular disorders (Pyykkö et al., 1991; Lekhel et al., 1997; Karlberg and Magnusson, 1998; El-Kahky, 2000; Yagi et al., 2000; Maurer et al., 2001; Eysel-Gosepath et al., 2016; Wuehr et al., 2018).

### 3.2 Vibration parameters

#### 3.2.1 Location

**Figure 3:**
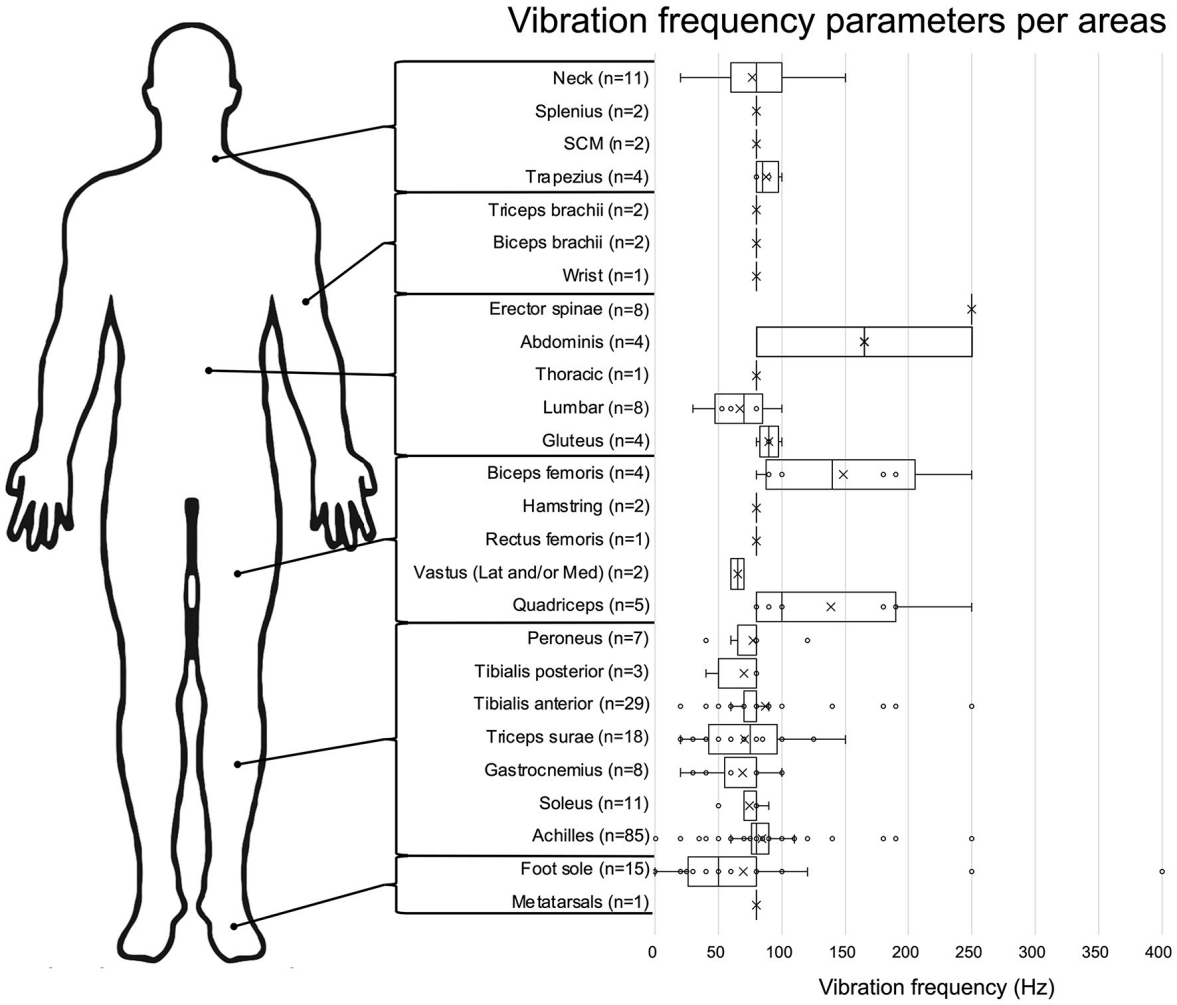
Vibration frequency parameters for each body area. Data are presented in box plot, with each vibration frequencies value represented by a circle and the means by an X. The number of studies assessing each frequency is presented next to the body area.

In general, studies targeted lower limb tendons or muscles to elicit VIB-PR (*n* = 137). As presented in Figure 3, most studies vibrated Achilles tendons (*n* = 85), followed by the tibialis anterior (*n* = 29). Cutaneous zones (under the feet) were stimulated in 16 studies. Trunk muscles were targeted in nine studies and upper limb structures (including tendons and muscles from the neck or arms) in 16 studies.

#### 3.2.2 Vibration duration

Duration varied greatly across studies, ranging from 0.03 s (dos Santos Fornari and Kohn, 2008) to 3,600 s (Lapole et al., 2012; Baudry and Duchateau, 2020). The mean duration for all duration values was 110.5 ± 463.3. Using SPSS to identify extreme values, the mean vibration duration across studies, once extreme duration values (*n* = 12; ≤ 2 s or ≥900 s) were removed, the mean duration was 36.5 ± 68.1 s, with 10 s and 20 s being the most frequent durations (*n* = 20 each), followed by 30 s (*n* = 17).

#### 3.2.3 Vibration frequency

Once again, this vibration parameter varied across studies, with a range going from 0.05 Hz (Maurer et al., 2001) up to 400 Hz (Inglis et al., 2002). As presented in Figure 3, vibration frequencies are usually around 80 Hz when targeting tendons/muscles (e.g., mean of 79.9 ± 35.4 Hz for the lower limb), while being more variable (mean: 69.8 ± 87.9 Hz) when stimulating skin mechanoreceptors under the foot.

#### 3.2.4 Vibration amplitude

**Figure 4:**
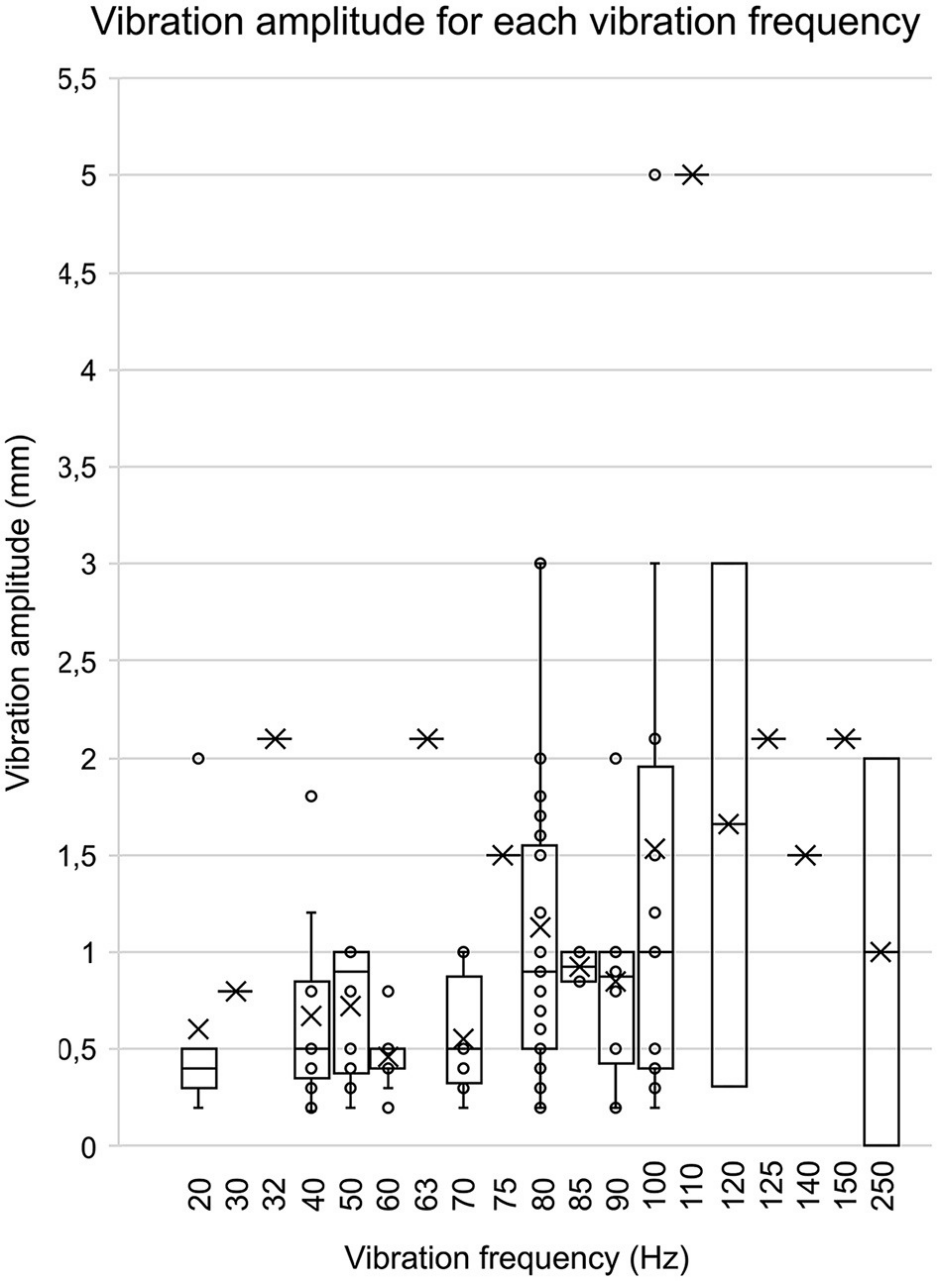
Vibration amplitude for each vibration frequency. This bar graph represents the associations between the various vibration amplitudes associated with their vibration frequency. The data were included in this graph only if both the amplitude and frequency were present in an article. The circles represent each amplitude value, and the mean is represented by the X.

When data were available (111 out of 147 articles), vibration amplitudes were extracted and pooled together. As shown in Figure 4, the majority of studies selected vibration amplitudes between 0.2 mm and 3.0 mm. Few studies opted for an amplitude lower than 0.2 mm [75 nm (Sacco et al., 2018); 200 nm (Lee et al., 2012, 2013; Martin et al., 2015); 1.8 mm (Oku et al., 2020)] or higher than 3.0 mm [5 mm (dos Santos Fornari and Kohn, 2008)]. The most frequent amplitude was 1.0 mm (*n* = 28), followed by 0.4 mm (*n* = 21) and 0.5 mm (*n* = 26). When looking at data comparing vibration amplitude from cutaneous zones (such as under the feet) to muscle/tendons (such as Achilles tendon or triceps surae), amplitude ranges for cutaneous vibration tend to be smaller (e.g., 0.2–1.8 mm for the feet, mean: 0.7 ± 0.6 mm) than for tendons/muscles (e.g., 75 nm to 5 mm for the Achilles tendon, mean: 1.3 ± 1.0 mm).

#### 3.2.5 Rationale for the selection of vibration parameters

Seventy-one out of 147 studies did not include any rationale nor reference to support the vibration parameters selected. Additionally, 11 studies cited a source to support their rationale, but it was located in the introduction instead of the methods. Out of the 65 studies that included any rationale in their methods, 18 studies cited either previous work from a member of the research team (*n* = 16) or referenced to unpublished pilot studies (*n* = 2). Finally, 53 studies supported their selection of vibration parameters from other studies, most frequently from the works of Roll and Vedel (1982) (*n* = 23) and/or Roll et al. (1998) (*n* = 23).

## 4 Discussion

The aim of this scoping review was to review the extent and range of populations and vibration parameters currently used in human research using the VIB-PR paradigm. By reviewing 147 original studies, our results demonstrated a high heterogeneity of methods and vibration parameters, and often a lacking or imprecise rationale to support those methodological choices.

Regarding the populations recruited, our results demonstrated that adults around 32.3 ± 12.7 years old tend to be more present in vibration studies. In support of this finding, mechanisms underlying VIB-PR are incompletely understood and still require fundamental research with younger and healthy participants (Kadri et al., 2020, 2023). For example, Kadri et al. suggested a new analytical method for precisely tracking spatiotemporal variables related to the center of pressure. They found new evidence that the precise time-course of postural reactions is in fact characterized by distinct phases that could be linked to different mechanisms of sensorimotor integration. Similar observations based on CoP displacements were also found in studies from different groups of researchers (Kavounoudias et al., 2001; Capicíková et al., 2006; Duclos et al., 2014). Also, Kadri et al. found that these patterns varied between and within individuals. The neurophysiological and behavioral correlates of these observations remain unknown (Kadri et al., 2020, 2023), calling for further fundamental work with neurologically and physically healthy individuals. In terms of knowledge gaps, however, our review highlights a greater need for studies testing VIB-PR with other populations than healthy adults. Collecting normative values from all age groups would help generalize findings to larger and more diverse populations and provide useful data for comparing with sub-populations having specific pathological conditions or personal characteristics.

Apart from healthy adults, the other subgroups identified by the scoping review were older adults and individuals with different pathologies or sociodemographic characteristics (*n* = 32 for older adults and *n* = 46 for all other studies combined). The main objective behind studies that tested these populations was to investigate how their specific particularities influenced VIB-PR observations. Older adults were the second most often studied population, probably because of the well-known age-related decline in sensory processing and postural control (Goble et al., 2011; Eikema et al., 2013, 2014). Thus, they present a relevant model to verify the validity of the VIB-PR paradigm and identify novel biomarkers of fall risks and sensorimotor dysfunctions (Abrahámová et al., 2009; Eikema et al., 2013; Bekkers et al., 2014; Ito et al., 2020). The same logic applies to pathologies affecting somatosensation, such as stroke (Kessner et al., 2016) or Parkinson's disease (Conte et al., 2013). As discussed above, studying how VIB-related mechanisms of sensorimotor processing are affected in these populations requires comparative data from age-matched controls, thus further supporting the importance of studying VIB-PR in older participants. For example, a study using VIB-PR to compare elderly fallers and elderly fallers suffering from Parkinson's disease suggested that it might affect specifically medio-lateral stability (Bekkers et al., 2014). Therefore, future studies could focus on this direction to better understand why medio-lateral stability declines in Parkinson's disease.

Regarding vibration location, our scoping review highlighted the fact that most studies focused on the ankle joint, and more specifically studied the Achilles tendon (Figure 3). This finding is not surprising since the ankle joint is known to generate strong postural reactions (Dettmer et al., 2013; Maitre et al., 2013a; Baudry and Duchateau, 2020; Kadri et al., 2020). Moreover, this joint is crucial for controlling the anteroposterior sway when standing in antigravitational postures (Arnold et al., 2009; Spink et al., 2011). One other possible explanation is that most seminal studies on VIB-PR (15 out of 20 studies) published before 2000 were performed on the Achilles tendon or muscles/tendons from the triceps surae. Therefore, it might be easier to compare their findings to previous results obtained from the same muscles/tendons. However, different patterns of VIB-PR have been observed when targeting the tibialis anterior tendons vs. the Achilles tendon (Kadri et al., 2023), which puts into question why only the Achilles tendons are chosen in most studies. Testing other musculotendinous structures would give a more complete picture of regulatory mechanisms involved in keeping balance in different directions and using different joints. For example, including peroneus muscles in future studies could be of great interest to study sensorimotor processing and postural control in the medio-lateral direction, especially in populations presenting higher risks of instability in this direction. Although conceptually linked, VIB-PR elicited when stimulating different locations distributed across the whole body (Figure 3) might not underlie the exact same mechanisms, nor are they testing the same neural loops. Our review stresses out the importance of knowing and filling these gaps of knowledge. In particular, only a few studies investigated VIB-PR elicited by stimulation of trunk, knee and hip muscles, despite their important role in postural control (Gribble and Hertel, 2004; Vuillerme et al., 2007; Bizid et al., 2009). In the same way, cutaneous afferent inputs from the foot sole or parts of the skin stretched when moving provide valuable proprioceptive information to regulate posture and movement (Inglis et al., 2002; Aimonetti et al., 2012). However, few studies focused on cutaneous vibration, or tried to disentangle the contribution of cutaneous receptors to the overall impact of VIB when targeting musculo-tendinous structures. The most eloquent demonstrations originate from studies published 40–50 years ago using anesthetized joint and skin afferents, and even exposed tendons during surgeries and found that VIB-induced illusory and proprioceptive effects were preserved. Although it underscored the core role of muscle afferents in proprioception, it cannot completely rule out the potential influence of joint and cutaneous afferents. More recent work found that certain types of cutaneous receptors such as Meissner or Pacinian corpuscles are responsive to frequencies of vibration within the ranges observed in the present review (i.e., 30–300 Hz) and that their activation by VIB influenced movement detection (Weerakkody et al., 2019). Of note, none of those previous studies having studied the complex interaction between skin and muscle afferents with VIB were realized in postural contexts. Altogether, this review highlights the paucity of evidence available on how cutaneous receptors contribute to the VIB-PR phenomenon (around 10% of the included studies), leaving once again a gap of knowledge to fill in order to better understand the regulatory mechanisms involved in keeping balance.

Finally, vibration frequency, amplitude and duration also varied greatly across studies (Figures 3, 4). There is good fundamental evidence supporting the impact of changing vibration frequency on the rate of muscle afferents depolarization and kinesthetic illusions (Roll and Vedel, 1982; Ribot-Ciscar et al., 1989). However, there are few evidence to support how changing the frequency actually impacts postural reactions, and all were obtained from healthy participants (Kavounoudias et al., 2001; Polonyova and Hlavacka, 2001; Schofield et al., 2015). For vibration duration, even when extreme values were removed, our results showed that the mean duration across studies was 36.5 ± 68.1 s, i.e., with almost 190% of variation. Choosing the most appropriate duration, even for other applications such as illusions, remains mostly arbitrary due to lacking empirical data (Seizova-Cajic et al., 2007). Recent work suggested that VIB-PR evolves dynamically over time, with at least a first phase of rapid postural reaction (first 2–3 s), followed by a later phase (at least 8 s) where postural control tends to restabilize (Capicíková et al., 2006; Duclos et al., 2014; Kadri et al., 2020, 2023). We would at least recommend choosing longer durations instead of shorter ones, in order to avoid missing relevant behaviors and underlying mechanisms of control. In Kadri et al. (2020, 2023), there were many participants who did not even reach their maximal amplitude of reaction after 10 s of vibration.

The specific effect of amplitude remains difficult to study. In our experience, technical specs of most vibration motors purchasable online show a non-linear relationship between vibration frequency and amplitude. Both parameters are modulated by only one controllable input: the strength of the electrical current. Increasing this electrical input increases both the frequency AND amplitude (up to a certain physical limit). In order to keep the amplitude fixed, the vibration device must be designed accordingly through advanced engineer skills. To our knowledge, this issue has never been raised before, despite several authors having reported using custom-made vibration apparatus (Ivanenko et al., 2000; Goble et al., 2011; Bekkers et al., 2014; Billot et al., 2015; Cofré Lizama et al., 2016). Some authors, however, used accelerometers to measure the amplitude (Courtine et al., 2007; Lubetzky et al., 2016). In general, illusory perceptions of movement were increased by using higher vibration amplitudes (Schofield et al., 2015; Taylor et al., 2017), possibly through a greater pool of spindle afferents activated, but this has yet to be demonstrated for VIB-PR. In any case, it would be appropriate to include, in future studies, a more complete description of the apparatus used and the general specs (e.g., type of electric motors, size of the apparatus,…). Furthermore, in their seminal work Goodwin recognized that their vibrator had an amplitude of about 2 mm but when applied on the participant, the amplitude was dampened to ~0.5 mm. From what we read in the included studies, none verified if the desired VIB parameters (amplitude, frequency) were conserved after having installed the vibrators on participants. One study however verified the impact of changing the tensile strength applied to the vibrator when strapping it the ankle and found that postural reactions were influenced when using higher vs. lower levels of tightness (Maitre et al., 2021). Unfortunately, they did not measure how different levels of tightness influenced VIB parameters, nor did they consider this effect as potentially explaining their results. Nevertheless, we believe that future studies should investigate this unexplored methodological factor.

This lack of knowledge about vibration parameters, especially when applied in postural contexts, might explain why a supporting rationale was not provided in almost half of the included studies. On the other hand, many based their methodological choices on two seminal papers from Roll and coworkers (Roll and Vedel, 1982; Roll et al., 1989). Of note, neither of these studies by Roll's group applied VIB in postural contexts. They rather investigated which sensory afferents best responded to VIB, which parameters (mostly frequency) resulted in the strongest activation of those receptors and how different parameters and modes of VIB application influenced kinesthetic illusions perceived by the participants. In both studies, it was demonstrated that perceived movement velocity reached a maximal value for a vibration frequency of about 60–80 Hz (or 70–80 Hz), with 80 Hz being the peak value. Once this “critical value” was exceeded, the individual response to the vibration was found to decrease (Roll and Vedel, 1982; Roll et al., 1989). These observations were obtained with an amplitude ranging from 0.2 to 0.5 mm with a duration never exceeding 20 s (Roll et al., 1989) or 0.5, 1 or 2 s (Roll and Vedel, 1982). Although such findings are highly relevant and impactful for the field, it remains uncertain if they can directly apply to VIB-PR since sensorimotor networks involved in the processing of VIB-induced afferents are probably not the same in postural vs. non-postural contexts. Moreover, as presented in Supplementary Data Sheets S1, S2 many studies (34 studies out of the 76 studies that included a rationale; 44.7%) cited at least one of these two papers to support their methodological choices, even if some of them used different parameters then those suggested by Roll and coworkers. A sound rationale to support the use of parameters exceeding the known limits of efficiency for depolarizing spindle afferents, such as around 80 Hz for frequency, was rarely provided. Therefore, our results underscore the critical need for establishing valid rationales to guide methodological choices in the field of VIB-PR. This knowledge gap is of the utmost importance; understanding the impact and standardizing vibration parameters are required for drawing adequate conclusions and enabling comparisons between studies.

As a first step toward this standardization, and in the absence of sound empirical knowledge of all parameters influencing VIB-PR, it is relevant to consider what parameters have been most often used in the field. The most “typical” vibration protocol found in the present review tends to generate a 20 s vibration at 80 Hz and around 1 mm of amplitude. This finding is similar to what has been described in a previous review focused on muscle vibration-induced illusions (Taylor et al., 2017) and is in accordance with previous results suggesting that muscle spindle afferents respond in an optimal “harmonic” 1:1 ratio at frequencies around 70–80 Hz (Roll and Vedel, 1982; Roll et al., 1989). As for the amplitude, previous results suggested that increasing vibration amplitude from 0.1 mm to 0.5 mm resulted in stronger, larger and faster vibration-induced illusions of movement (Schofield et al., 2015). Finally, >20 s durations might be necessary to encompass the most important mechanisms and behaviors at play during vibration applications. However, the short- and long-term neuroplastic adaptations of a sustained application of vibration has not been sufficiently addressed. The available evidence at least encourages future research to choose an appropriate duration based on the specific mechanism/behavior being targeted. For example, if eliciting tendon reflexes is what the research team is aiming for, short vibration bursts of 0.03 s have been shown to be sufficient (dos Santos Fornari and Kohn, 2008). On the other hand, if a research team aims to describe the time course of CoP displacement, induce lasting neuroplastic changes or investigate sensory-reweighting mechanisms necessary for reaching postural re-stabilization when a sensory source is no longer recognized as appropriate (Kadri et al., 2020, 2023), a longer vibration duration such as >10 s should be considered.

Our review has some limitations inherent to the study design. First, the quality of the included studies was not assessed in order to highlight some biases. However, scoping study usually does not seek to assess quality of evidence (Arksey and O'Malley, 2005). Also, due to the nature of a scoping review, our results are not weighted according to the studies' quality. Future studies should, therefore, focus on synthesizing the results in a systematic review and recommend specific vibration parameters. Until then, we suggest that results from studies using vibration should be discussed with caution, especially regarding the purposes of the research and/or when conducting clinical assessments in clinical setting because of the variation inherent from the vibration parameters applied.

## 5 Conclusion

This scoping review provided a comprehensive description of the population recruited and parameters used for vibration protocols in current studies with humans. This study is a first step toward improving the standardization of VIB-PR methodology to ensure a higher impact and, eventually, transfer to clinical applications. Despite a large number of studies, there are still important gaps in knowledge that need to be filled, especially for younger populations or pathological conditions, as well as vibration amplitude and duration parameters. Furthermore, we strongly encourage future work to appropriately support each methodological choice regarding VIB-PR parameters and protocols, based on the best available evidence and sound scientific rationales.

## Data availability statement

The original contributions presented in the study are included in the article/Supplementary material, further inquiries can be directed to the corresponding author.

## Author contributions

MB-C: Conceptualization, Formal analysis, Methodology, Writing – original draft, Writing – review & editing. M-PP: Conceptualization, Methodology, Writing – review & editing. RS: Conceptualization, Writing – review & editing. L-DB: Conceptualization, Funding acquisition, Resources, Writing – review & editing.

## Supplementary material

Supplementary Data Sheet S1 Study extraction data.

Supplementary Data Sheet S2 Methodological rationale data.
